# Political and Institutional Influences on the Use of Evidence in Public Health Policy. A Systematic Review

**DOI:** 10.1371/journal.pone.0077404

**Published:** 2013-10-30

**Authors:** Marco Liverani, Benjamin Hawkins, Justin O. Parkhurst

**Affiliations:** Department of Global Health and Development, London School of Hygiene and Tropical Medicine, London, United Kingdom; Consejo Superior de Investigaciones Cientifics, Spain

## Abstract

**Background:**

There is increasing recognition that the development of evidence-informed health policy is not only a technical problem of knowledge exchange or translation, but also a political challenge. Yet, while political scientists have long considered the nature of political systems, the role of institutional structures, and the political contestation of policy issues as central to understanding policy decisions, these issues remain largely unexplored by scholars of evidence-informed policy making.

**Methods:**

We conducted a systematic review of empirical studies that examined the influence of key features of political systems and institutional mechanisms on evidence use, and contextual factors that may contribute to the politicisation of health evidence. Eligible studies were identified through searches of seven health and social sciences databases, websites of relevant organisations, the British Library database, and manual searches of academic journals. Relevant findings were extracted using a uniform data extraction tool and synthesised by narrative review.

**Findings:**

56 studies were selected for inclusion. Relevant political and institutional aspects affecting the use of health evidence included the level of state centralisation and democratisation, the influence of external donors and organisations, the organisation and function of bureaucracies, and the framing of evidence in relation to social norms and values. However, our understanding of such influences remains piecemeal given the limited number of empirical analyses on this subject, the paucity of comparative works, and the limited consideration of political and institutional theory in these studies.

**Conclusions:**

This review highlights the need for a more explicit engagement with the political and institutional factors affecting the use of health evidence in decision-making. A more nuanced understanding of evidence use in health policy making requires both additional empirical studies of evidence use, and an engagement with theories and approaches beyond the current remit of public health or knowledge utilisation studies.

## Introduction

In the past two decades there has been a tremendous increase in expenditure on global health research. The Global Forum for Health Research, for example, reported that international funding for this has risen from US$30 billion in 1986 to an estimated $163 billion in 2005 [1]. Despite this growth, concerns remain that health policy and practice are not adequately informed by the best available evidence, and that research findings may take too long to be incorporated in policy processes. As former World Health Organization (WHO) director Lee Jong-wook stressed “there is a sense that science has not done enough, especially for public health, and there is a gap between today’s scientific advances and their application: between what we know and what is actually being done” [2]. In this context, a growing body of literature has begun to study the use of evidence in public health policy-making through empirical and conceptual analyses, attempting to identify technical barriers and facilitators that may influence the timely uptake of research findings [3], [4]. In addition, a diverse range of initiatives has attempted to bridge gaps that are seen to exist between research and policy [5], including regional networks to link researchers and policy makers [6], user-friendly data repositories [7], guidelines for drafting and using evidence-informed policy briefs [8], and training of decision makers on how to use systematic review information [9].

However, within research and policy communities there has been increasing awareness that getting research into policy is not only a technical matter of knowledge translation and exchange, but also a political challenge [10]. A report by the Alliance for Health Policy and Systems Research, for instance, noted that: “Policy making is a complex and essentially political process that is influenced by several factors (…) Recognition and understanding of decision-making processes and factors that influence the process can increase the potential for inserting research information into the process” [11].

Outside the field of public health, the discipline of policy studies has long explored the decision-making process, illustrating how different political systems shape the capacity of governments to develop effective policies. Key political system features include the territorial structure of the state – whether it is a unitary (centralised) or a devolved (federal) state [12] – the level of democracy (the degree of political pluralism and freedom), and the role of the bureaucracy (in particular their degree of control over the policy advice given to decision makers) [13], [14].

It is also well established in social and political science that decision-making processes involve tradeoffs between competing interests and values [15] and, as such, analyses of policy making must pay attention to the ways in which a given issue is politicised. Even within a single sector (such as health), policy decisions can be contested in numerous ways. All decisions will have implications for sectoral budgets and priorities, and will imply certain opportunity costs; but health policy issues are also likely to involve social considerations beyond clinical outcomes alone - such as questions of equity, justice, or morality – all of which can influence decision making around any given body of health evidence [16].

While social and political scientists have long considered the nature of political systems, the role of institutional structures, and the political contestation of policy issues as central to understanding decision making, these issues remain largely unexplored by scholars of evidence-informed policy making. It remains unclear, for example, how the political nature of a given health issue might affect the use of relevant evidence (for example, when an issue is morally contested or impinges on powerful economic interests). Nor is there significant understanding of whether particular state structures or institutional bureaucratic arrangements may facilitate or impede the use of evidence for health decision making.

To advance our understanding of these questions, we conducted a systematic review of empirical studies that examine the complex interface between politics, policy, and the use of evidence. Previous systematic reviews have been undertaken on aspects of health research utilisation, but these have primarily focussed on technical aspects of knowledge translation including barriers to evidence use, and ways to overcome them. Past reviews have addressed, for example, policy makers’ perceptions of their use of evidence [17], the effectiveness of interventions designed to increase the use of research in clinical practice [18], and the usefulness of systematic reviews for health care managers and policy-makers [19]. This journal has also recently published a systematic review of the use of evidence in settings with universal health care systems, which concluded that “organisational, political, and strategic factors” restricted the use of evidence in public health decision making. That paper further noted that most systematic reviews of policy studies “tend to overlook the impact of political and institutional context” [20]. By contrast, our systematic review explicitly addressed the political nature of decision making, seeking to identify what is currently known about the ways in which political factors shape the uptake and use of evidence in health policy making. In order to capture the variety of potentially relevant themes, we maintained a broad understanding of ‘politics’ that goes beyond state institutions and party politics, to include political culture, ideologies, and informal arrangements at both the national and international level [21]. For the same reason, we reviewed studies that focused both on specific health conditions as well as broader health concerns such as access to care, the organisation of health services, and the social determinants of health. However, as our focus was specifically at the policy level, we discarded studies that did not focus on public health issues, such as studies that examined the uptake of evidence-based guidelines within routine clinical practice.

## Methods

### Search Strategy

The methods of the analysis and inclusion criteria for the study were specified in advance and are documented in the attached protocol (Text S1). Eligible studies were initially identified using the following health and social sciences databases: Global Health, Healthcare Management Information Consortium (HMIC), International Bibliography of the Social Sciences (IBSS), MEDLINE, PubMed, Social Policy and Practice, and ISI Web of Science. No limitations were placed on language, although all search terms were in English. Whilst there is a literature on evidence use in policy making that predates the period reviewed [22]–[25], we limited our search to materials published after 1990 for two principal reasons. First, we were mainly interested in exploring the contemporary context of health policy making. Second, the ‘evidence based medicine’ movement, which gave an increased impetus to demands for evidence informed health policy, is widely seen to have taken shape in the 1990s [26] (for example, with the creation of the Cochrane collaboration in 1993). The listed databases were searched in May 2012, using two broad query strings that enabled comprehensive searches of relevant material:

1. Health polic* AND (research OR evidence) AND (governance OR institution* OR polit*);
2. Evidence-based AND (health* OR medicine) AND policy AND (governance OR institution* OR polit*)

Internet search engines such as Google and Google Scholar were also used to identify material which was not published in journals or available online (e.g. monographs and book chapters). The British Library catalogue was searched to identify relevant titles which met our inclusion criteria. Individual websites were then reviewed for grey literature, selected on the basis of their relevance to the subject and on recommendation from key individuals working in the field. These included the online repositories of the Program in Policy Decision Making at McMaster University, the Centre for Evidence & Policy at King’s College, the WHO Evidence into Policy Network (EVIPNet), and Evidence to Policy Initiative (E2Pi) at the University of California at San Francisco.

Finally, manual literature searches were undertaken in the online archives of the following journals from 1990 until May 2012: *BMC Health Services Research*; *Evidence & Policy*; *Health Policy*; *Health Policy & Planning*; *Journal of Health Politics, Policy, and Law*; *Milbank Quarterly*; *Social Science & Medicine*; *Health Research Policy and Systems*. The bibliographies of all included text were also reviewed for further relevant citations. Articles were downloaded to an *Endnote* database and duplicates were removed. All remaining references were then transferred to a *Mendeley* database to facilitate collaboration during the screening and data extraction phases.

### Selection of Studies

**Figure 1:**
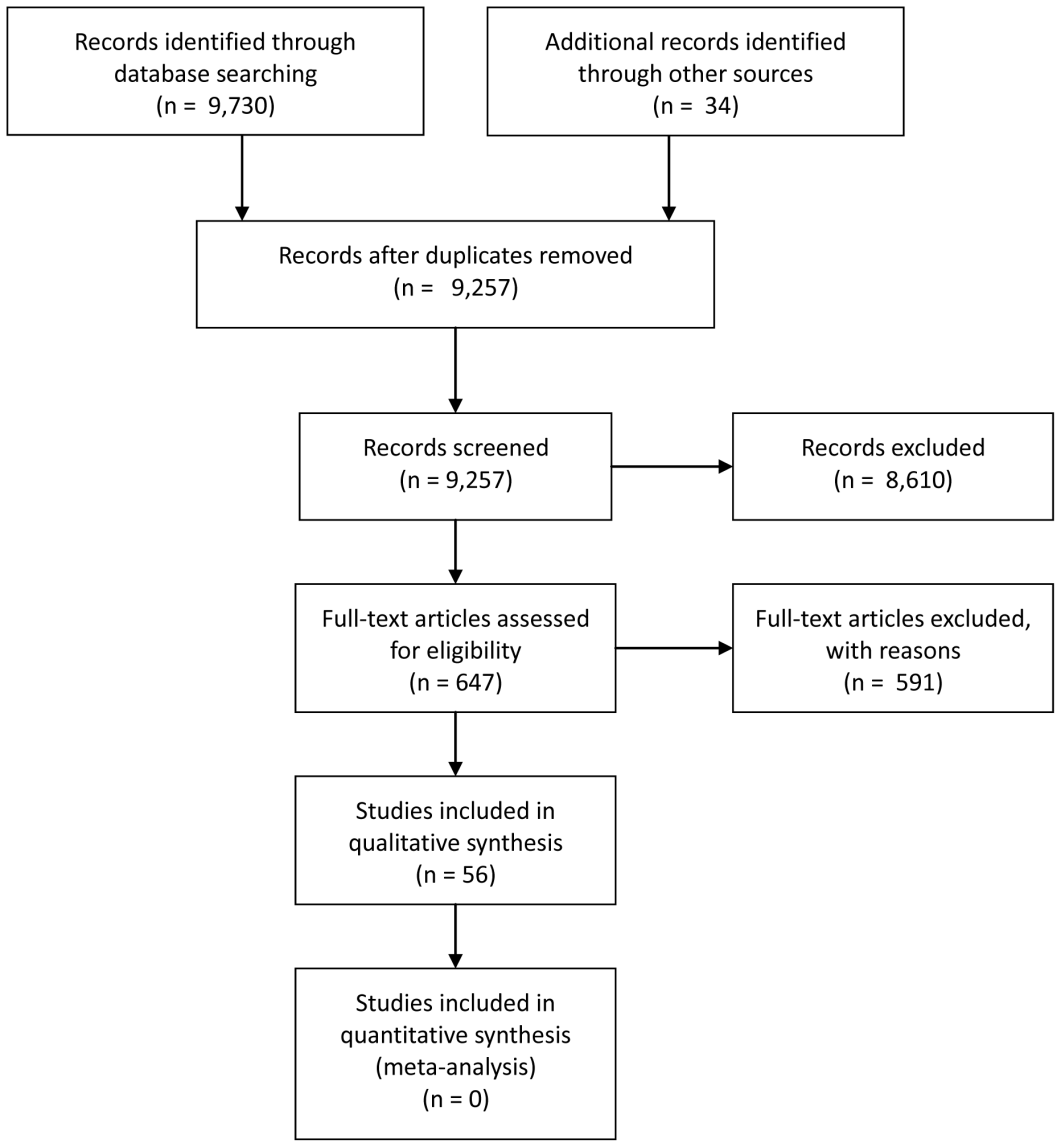
PRISMA flow diagram.

Electronic searches yielded 9,730 items. A further 34 items were identified through searches in journal archives, recommendations of colleagues, and through following up of references found in the included articles. After removing of duplicates, 9,257 unique studies remained. The first and second author conducted a preliminary screening of all retrieved studies on the basis of titles and abstracts. 647 articles met the inclusion criteria and were retained. The full texts of these were obtained and assessed for relevance (Figure 1).

Given the dearth of specific studies on the politics of evidence-based health policy, we adopted an inclusive approach throughout the selection process. It was decided to include works whose aims were tangential to the central focus of our review, but which presented and discussed relevant findings. However, papers that provided a description of wider political and institutional contexts, but did not explore the ways in which such contexts may have shaped the use of evidence were excluded. We also excluded all contributions that were not supported by empirical data, such as commentaries, letters, and conceptual frameworks. Finally, all three authors discussed and refined the inclusion criteria at several stages of the process and independently reviewed the final list of articles included.

### Data Extraction and Analysis

The first and the second author extracted data from included studies using a uniform data extraction tool (Table S1). In addition to information on the study characteristics (e.g. methodological approach, data sources, country focus, health issues), relevant findings were identified and analysed according to three research questions derived from political studies theories and designed to address the gaps identified above in the existing literature on evidence to policy processes:

1. Whether the study analysed features of political systems that influenced the use (or neglect) of evidence for health policy;
2. Whether the study analysed institutional mechanisms that influenced the use (or neglect) of health research;
3. Whether the study analysed other contextual factors that contributed to the politicisation and contestation of health evidence.

The relevant material extracted from the included studies was tabulated and organised according to the three overarching questions noted above. Within these broad thematic areas, collected data were combined according to recurrent issues that emerged from the studies themselves and presented by narrative review [27]. Given the diverse and generally non-cumulative nature of selected studies, it was not possible to incorporate all extracted information in the presentation and discussion of results. However, Table S2 provides the comprehensive collection of research findings.

## Results

After full-text review, 56 studies were selected for inclusion. They focused on a variety of countries, settings, and health issues. Most included studies utilised qualitative methods such as semi-structured interviews and critical analysis of policy documents, although different approaches and sampling criteria were used. Most studies examined single cases of research use in one country, but 12 studies included multiple country comparison or data and 4 studies provided comparative analysis of different cases of research utilisation in the same country. With few exceptions, selected studies can be situated in the field of public health and were primarily driven by practical concerns with health care and health services. Indeed, 35 out of 56 studies were published in health journals, while the majority of the remainder were published in public policy journals. In most contributions the analysis of political and institutional elements was often subordinate to other research objectives, such as the identification of technical barriers to, and facilitators of, the use of evidence or descriptions of the ways in which evidence was used to inform specific health policies or programmes. Only 6 studies were judged to have explicitly engaged with political theories or concepts [28]–[33]. In the majority of cases, when issues relevant to our research questions were included in the studies, often there was only passing mention of these factors, and the points raised were rarely analysed in depth. However, due to the multi-disciplinary nature of our inquiry and desire for maximum coverage, we opted to include any studies which might provide insights for each of our research questions.

### Political Systems

The first of the key questions we set out to explore is whether any links could be found between the use of evidence for health policy and key features of different state political systems (such as the structure of decision making authority, or level of public participation and democracy). We identified 4 studies that explicitly addressed this question, all of which were concerned with the implications of centralised governance. One study explored decision-making processes at the UK National Health Service (NHS) prior to the reforms of the late 1990s [30]. This study suggested that centralised political systems are likely to be less open to the uptake of research findings than de-centralised systems. The concentration of power was found to prevent pluralistic debate and thus the need for evidence to support competing views. By contrast, it was argued that in countries in which policy is made through ad-hoc, issue specific coalitions, such as the United States, and in federal systems in which policy is made at the provincial level, “there is more need for research as legitimation or ammunition” to justify policy decisions and defend them against the criticisms of opponents.

Another study examined the failure of the British government to handle risk communication during the public health crisis of bovine spongiform encephalopathy (BSE) in the late 1980s. Having analysed the political and institutional context in which decisions about scientific evidence were made, this study found that a centralised system in which government agencies control expert advice with little public oversight is more vulnerable to the pressure of expert interest groups in decisions around evidence use [29]. Other studies documented how financial and corporate interest groups exert pressure on decision makers either to take up or ignore research findings, based on their commercial interest [34]–[37]. One study argued that the lack of pressure from organised lobbies in Laos facilitated the use of evidence for health policy on essential medicines in this country [38]. However, none of these studies explicitly examined whether differing political or institutional contexts may be more or less susceptible to such influences. Finally, one study on the use of health research in Mexico found that hierarchical management of information in a centralised system may prevent research results to arrive at operational levels, where they could have greater impact and usefulness [37].We did not identify any studies that analysed in depth the use of health evidence in one-party or authoritarian states. However, a case study on the development of health insurance in rural China illustrated the value of having a broker in the research team who understands political practices and thus can propose solutions that are compatible with the wider policy agenda of the central government [39]. The same study also noted that international experts and organisations have played a key role in providing research input for radical policy change in this country, given the lack of autonomy of domestic bodies that support health and health services research.

We found no studies that systematically explored the effects of democratisation on the use of evidence, but the findings of some studies which noted the importance of changes in regime are relevant to this question. Two studies reported that the shift to parliamentary democracy in South Africa in 1994 created a new governance model that was more open to the uptake of research findings, as academic researchers were appointed to managing positions in the National Department of Health and new research institutes were established to support policy making [40], [41]. Similarly, another study reported that the establishment of a democratic government in Uruguay after the end of the military dictatorship in 1983 contributed to the enhancement of research capacities for infectious disease control and a culture of evidence-based policy. For example, new funding was allocated to science and the reconstruction of research facilities, which had been reduced drastically during the totalitarian regime [42]. However, none of these studies provide an analysis of specific political mechanisms that facilitated these developments. In principle, health research institutes could also have been appointed during the previous regimes. Indeed, a comparative study of the “market for evidence” in child health policy found that the nature of the political system (e.g. democratic or autocratic) is not necessarily a key factor in influencing the use of evidence in policy making [43]. Other studies suggested that potential biases in the use of evidence may result from processes of democratic deliberation, including opportunistic use of evidence to delay decision-making [44], to legitimate particular policy positions or to discredit opponents in political debates [45]–[48]. One study on the policy process around the implementation of a bowel cancer screening programme in Australia noted that evidence may become more contested during electoral campaigns, as underlying tensions between stakeholders who control the selection of evidence for policy are likely to be amplified [49]. Finally, one study on health policy making in Australia found that issue polarisation dictates the extent to which research or researchers are used technically or politically [50].

### Institutional Mechanisms

Our second area of analysis was to identify empirical studies of how different institutional mechanisms (such as the structures, processes, or regulations followed by bureaucracies) shape the use of health evidence. While more studies were identified which addressed this issue than the theme of political systems, many studies cut across a wide range of topics, providing a variety of disparate findings.

One study on policy for health inequalities in the UK, for example, found that the division of responsibilities within government bureaucracies limited the use of evidence, arguing that “individual civil servants are compelled to focus on small, specific areas of policy activity, making it extremely difficult for them to engage with ideas beyond their immediate area of responsibility” [32]. Two further cases illustrated how institutional ‘silos’ could limit evidence consideration for complex health issues that require multi-disciplinary evidence and horizontal thinking across sectoral boundaries, such as in a bowel cancer programme in New Zealand [51] and HIV/AIDS policy in Cambodia [52].

Institutional path-dependency was found to be another important hurdle to unbiased or systematic evidence use. A study of WHO’s management of the 2009 H1N1 pandemic suggested that previously chosen policy responses are likely to be prioritised over alternative evidence-informed options, especially when the health issue is surrounded by scientific uncertainties [28]. Another study of HIV policy in Tanzania suggested that path-dependency may result from self-perpetuating mechanisms of organisational practices. This study found that evidence indicating a need to address upstream structural drivers of HIV was not acted upon because, “any shift in prevention priorities would imply taking funds away from some organisations and directing them to others - putting at stake the former’s institutional existence” [53].

Another institutional challenge noted in three studies was how high turnover of staff in health departments could undermine the systematic use of evidence for policy or planning [32], [49], [53]. One of these studies noted that frequent replacement of civil servants in UK health departments resulted in a lack of ‘institutional memory’ and the recycling of old but seemingly novel ideas, thus “creating the illusion that research is informing policy far more than it is” [32].

We found a number of studies which included some institutional analysis of bodies outside government departments of health which are tasked with facilitating evidence utilisation and knowledge brokering. A comparative study of nine Latin American and three European countries, for example, found that the use of evidence from economic evaluations for health policy decisions was increased in countries (such as the UK and Portugal) which had formally-mandated organisations to provide such evidence [54]. Other studies reported that the existence of organisational and institutional structures for knowledge brokering and exchange can greatly facilitate the uptake of evidence [6], [55]–[58].

Works focussing on non-governmental evidence-providing bodies also pointed to how personal connections between knowledge brokers and decision makers can be an important factor increasing the uptake and use of evidence in both high-income [55] and developing countries [39], [59]. One study, however, found that in three high income countries (US, UK, and the Netherlands) greater attention was paid to the reputation and professional legitimacy of institutions charged with the production or use of evidence, while in three middle-income countries (China, South Africa and Chile) it was personal relationships with experts that appeared essential [60]. Another comparative study in Bolivia, Cameroon, Mexico, and the Philippines found that the existence of a strongly motivated senior health official who championed evidence-informed concepts was essential in driving the policy process [61].

### The Political Nature of the Health Issue

Our final theme of analysis was to explore other contextual factors that may influence and politicise the use of evidence in health policy making. A set of studies were identified that analysed how existing normative positions including: values and moral convictions [31], [48], [62]–[64], religious and cultural identity [65], [66], or nationalism [46], [67], [68] can bias the selection or interpretation of evidence used for health policy development. One study showed how the understanding of evidence on the advantages of breastfeeding in the US may have been biased by highly entrenched values and beliefs around motherhood and infant rearing [64]. Another study in Ghana similarly noted that evidence on the health effects of procedures such as male circumcision were influenced by religious and cultural views [66]. Two further studies found that resistance to Western concepts of science, grounded in ideas of nationalism and cultural specificity, helped to explain why former South African president Mbeki rejected evidence on the causal link between HIV and AIDS [67], [68].

Several further studies noted how the alignment of health issues with existing political priorities in a country can affect the use of evidence. A study on health policy in China reported that research findings on the impact of health expenditure on rural poverty had a direct impact on policy because they reflected already existing concerns of policy-makers about economic growth and poverty reduction [39]. Further case studies from the UK [69], [70], the US [71], Uganda [46], and a comparison of three African countries [72] also suggested that the objectives of the wider policy agenda may influence how and what evidence is used. Related to the above, 4 studies noted the importance of the discursive framing of evidence in shaping its use in policy debates [31], [36], [73], [74]. One study, examining the policy debate on tobacco control in New Zealand, concluded that ’exceptional storytellers’ are needed to drive the process of policy making [36]. Another study illustrated that successful political uptake of evidence-based solutions may require “a shift in meaning of the idea or, at the very least, a more flexible construction of the idea” [33], especially if policy strategies overtly conflict with the dominant political ideology.

An article from Canada further suggested that policy decisions about the content of an intervention may be more amenable to the influence of research than broader or more conceptual decisions with multiple social implications [75]. Another study from Israel made a similar distinction, finding that evidence is more likely to be used for ’second order’ decisions (how to do something), rather than for ‘first order’ decisions (whether to do it), although it found first order decision which were technical in nature were also likely to be informed by data [76].

Several studies from lower-income settings noted that when a health issue is particularly important to international donors, this too can impact on the use of evidence, including the ways in which research is prioritised and conducted, and how findings are interpreted and used to inform policy decisions. Donors tended to promote interventions with strong evidence bases, but they do so in ways that may neglect local context, needs and capabilities [43], [52], [53], [77], [78].

Finally, the tensions between national and international concerns and the influence of national as opposed to international evidence was also a theme that appeared in some included studies. A study from the Netherlands found that there was a growing reliance on international rather than national data, raising concerns over the relevance of such research to specific country needs, the ease of access to findings, and the perceived legitimacy by local policy makers [79]. However, a seven-country comparative study of vaccine adoption [80] and a health insurance study from China [39] both reported that the use of local data had a stronger impact on national policy process than evidence from international sources.

## Discussion

This review arose from recognition of the importance of political factors in shaping the use of evidence to inform health policy processes, yet the limited explicit engagement with relevant theories in the literature on evidence-informed health policy. We structured our analysis around three analytical themes emerging from social and political sciences as explaining policy outcomes: the features of state political systems, the institutional structures and arrangements of bodies using evidence, and the politicisation of specific issues. A stand-out finding from our review was that little empirical work has been done explicitly analysing these issues, with many included studies dealing with them only indirectly.

The systematic review approach, however, enabled us to identify the political and institutional factors that have so far been identified in the existing literature as important in shaping the uptake of evidence for health policy making. These factors include concentration of power and political centralisation, levels of democratisation, institutional mechanisms and processes, turnover of staff in government bodies, the influence of donors and external organisations, the pressure of wider policy strategies and political cultures, as well as the alignment of evidence with predominant values or existing political agendas. Results also indicate that processes of evidence-based policy making often involve a complex interplay of these factors, both at the national and international level.

Nevertheless, our understanding of these issues remains patchy and inconclusive. The selected studies do not constitute a clearly defined body of research, developed around shared debates, research questions or theoretical approaches. While many studies relied on theories of knowledge utilisation, these were generally applied to support and guide empirical work rather than using empirical works to test or refine specific theories. Most notably, despite the fundamentally political nature of decision making processes, the extensive literature on political institutions, and the highly contested nature of many health issues, very few works could be identified which explicitly applied policy science perspectives to understand the use of evidence in health policy making. As a result, the analysis of political and institutional influences was limited, with no significant consideration of key issues such as the influence of party systems, the relations between different branches of government, power imbalances and hierarchies, and questions of political legitimacy.

Furthermore, the highly contextual nature of the policy process points to the need for comparative case study analysis to facilitate theory development [81]. For example, comparative approaches would be particularly fruitful to test hypotheses about the effects of more or less centralised political or administrative systems on the use of evidence. A number of multi-country studies relevant to our issues of interest were included in our review, but we found no studies that explicitly tested hypotheses in such a way and only 5 studies in which the use of a comparative approach was associated with a more explicit engagement with political and institutional analysis [33], [43], [66], [78], [82]. Similarly, we found only 4 studies that examined comparatively the effects of political and institutional change on evidence use practices within the same country [37], [42], [69], [74].

Finally, we conducted this literature review according to systematic, and therefore replicable, criteria for data collection and inclusion [83]. However, the application of these standards for analysis and data extraction was not always straightforward. Quality appraisal posed particular challenges, given the diversity of selected studies, the multitude of disciplinary approaches (with no standard of practice on methods or method reporting) and the lack of universal standards for the definition of ‘quality’ in qualitative research [84], [85]. In light of this, we decided to include all empirical studies that met our inclusion criteria as long as they documented their data sources, including those in which methods of data analysis were not clear. We shall note that similar challenges have been raised in past discussions around the difficulties in identifying clear criteria by which qualitative research should be assessed in systematic reviews [86].

Despite these limitations, our review contributes to improved recognition of the political dimension of health evidence utilisation, and can thus stimulate critical thinking about current policy debates. In particular, the perceived need to ‘bridge the gap’ between research and policy has encouraged the development of technical recommendations to facilitate research uptake; with the universal value of using the research often taken for granted. For example, some consensus has emerged in recent years about the benefits of closer interactions between researchers and policy-makers, from the early stages of research processes [4], [87]–[89]. One study concluded that “effective use of evidence requires a move away from a simple characterisation of ‘two communities’ and a new view of research being generated jointly in partnership with researchers and decision-makers” [90]. As recent initiatives illustrate [5], such interactions have the potential to promote the generation of policy-relevant research in different contexts. However, such efforts typically overstate the ability of better linkages between researchers and policy makers to facilitate evidence use or overcome political contestation, as was documented by an early evaluation of research utilisation at the UK Department of Health [22]. Given the high turnover of politicians and administrators in key decision making positions, training or linking of individuals may not produce sustained improvements in evidence use. Rather, establishment of institutional processes and procedures setting the standards for evidence use over time may be needed.

Furthermore, our approach raises concerns about the public health community’s tendency to depoliticise the idea of evidence use, evaluating policy making processes simply by whether, how much, or how quickly pieces of evidence are taken up by policy makers. Such an approach can obscure the complexity of the policy making process in which policy makers must evaluate competing social outcomes and value systems in ways which are accountable to local populations. Simply calling for policy to be ‘evidence based’ encourages decision makers to prioritise those issues where a large or more coherent body of evidence is available (such as for clinical treatments which have been evaluated in experimental trials) as opposed to more complex social and structural interventions to prevent ill health for which it is not so easy to identify direct causal mechanisms or gather evidence of immediate effect [16], [91].

Our review also suggests that an unreflective acceptance of over-simplified concepts of ‘evidence based policy’ is not conducive to good governance practices. For example, political pressures may encourage a selective use of evidence as a rhetorical device to support predetermined policy choices or ideological positions, or may delay decision-making on contentious issues while less contentious topics with clearer, uncontested evidence bases are followed. Such biases are likely to be amplified when there is a lack of institutional capacity or structures able to provide competent and independent scientific advice. However, empirical studies indicate that even institutional bodies that operate according to the most rigorous procedures may be vulnerable to distortions and/or the influence of interest groups [29], [92]. Thus, more attention needs to be paid to the specific kinds of evidence used at different stages of the policy making process, and the ways in which different political and institutional drivers may contribute to more or less appropriate evidence utilisation.

## Conclusions

This review identified a number of key issues at the interface of health policy, politics, and evidence use. With a greater awareness of the politics of evidence-informed health policy, more specific and explicit questions must be asked about the effects of different political factors impacting on evidence use in policy making and their interdependencies. What kind of institutional arrangements are more likely to facilitate a ‘good’ use of evidence in different political contexts? Are decisions seen as more legitimate if evidence is used to inform decision-making? How can these be evaluated? What can be done to reduce the biased and opportunistic use of evidence for political ends? To what extent can evidence-based guidelines promote policy change across different political and institutional settings?

Improved understanding of questions such as these requires the in-depth analysis of case studies of research uptake, with careful consideration of the institutional and organisational arrangements that may influence the selection and use of evidence. The work of Kogan and Henkel in the 1980s, for example, undertaking a seven year evaluation of the UK Department of Health’s use of evidence [22], has continued to inform discussions of evidence use today [23], [93] as it is a rare example of in-depth empirical work on the subject. Similarly Carol Weiss’ pioneering work in the 1970s and 80s on the multiple meanings of research utilisation [24], [25] continues to set the basic conceptual framework for many contemporary studies looking at evidence uptake or knowledge transfer [94]–[96]. At the same time, however, exploring the complexity of the multi-dimensional links between research and policy making requires innovative thinking and an engagement with theories and approaches beyond the current remit of public health and knowledge utilisation studies. Relevant insights are likely to be drawn from fields such as political and institutional theory, public administration, political sociology, and science and technology studies. There is a rich body of comparative studies of science policy and environmental risk regulations, for example, which could provide useful methodological tools and theoretical insights to this task [97]–[99], but which have not yet been applied to current policy questions in public health. Comparative analysis of evidentiary practices would be particularly useful to discern patterns and regularities across different institutional and political environments. In turn, the analysis of such patterns would provide empirical foundations for a more theoretically informed understanding of these issues. This will be a major research challenge, given the complexity of decision-making systems, and the important role of intervening variables in the wider socio-cultural contexts. Yet, it is a challenge worth taking up if we are to develop institutional structures and mechanisms that can work to ensure an effective and appropriate use of evidence in health policy and practice.

## Supporting Information

Table S1 **Data extraction form.**
(DOCX)

Table S2 **Characteristics of included studies.**
(DOCX)

Text S1 **Research protocol.**
(DOCX)

Checklist S1 **PRISMA checklist.**
(DOC)
