# Supplementary material for: Political and Institutional Influences on the Use of Evidence in Public Health Policy. A Systematic Review
Source: PLoS One. 2013 Oct 30;8(10):e77404. doi: 10.1371/journal.pone.0077404 (PMC3813708; doi:10.1371/journal.pone.0077404)
Supplement: Table S1 — Data extraction form. (DOCX) [file pone.0077404.s001.docx]

| **Data Extraction Tool** | |
| --- | --- |
| *Data to be extracted* | *Notes* |
| **Details of publication** | |
| Bibliographic details |  |
| Timeframe | [when was the study conducted?] |
| Methodology (or methodologies) used | [e.g. qualitative, quantitative, mixed, comparative] |
| Data source(s) | [e.g. surveys, interviews (including achieved sample), focus groups, documentation, secondary data sets, observations] |
| Literature in which it is situated |  |
| Explicit use of (explanatory) theory? |  |

| **Research focus** | |
| --- | --- |
| Country case(s) |  |
| Health issue(s) |  |
| Informants | [e.g. ministers, hospital managers, policy entrepreneurs, scientists, administrators, ‘knowledge brokers’] |

| **Summary/key points** |
| --- |
|  |

| **Relevant themes and findings** |
| --- |
| *Does the study provide any analysis of features of political systems that influenced the use (or neglect) of health research? If yes, can you give details?* |
|  |
| *Does the study provide any analysis of institutional issues/processes and government structures that influenced the use (or neglect) of health research? If yes, can you give details?* |
|  |
| *Does the study provide any analysis of other contextual factors that contributed to the politicisation and contestation of health evidence? If yes, can you give details?* |
|  |

| **Further comments** |
| --- |
|  |
