# Supplementary material for: Political and Institutional Influences on the Use of Evidence in Public Health Policy. A Systematic Review
Source: PLoS One. 2013 Oct 30;8(10):e77404. doi: 10.1371/journal.pone.0077404 (PMC3813708; doi:10.1371/journal.pone.0077404)
Supplement: Table S2 — Characteristics of included studies. (DOCX) [file pone.0077404.s002.docx]

| **Study** | **Research Objectives** | **Setting** | **Health issue** | **Data sources** | **Relevant findings** |
| --- | --- | --- | --- | --- | --- |
| **Abeysinghe 2012** | To provide a critical analysis of WHO’s management of the H1N1 pandemic. | Global health policy. | H1N1 | Policy documents; press releases | Institutional processes were pivotal in a decision-making context characterised by scientific uncertainties. The study found that the WHO was path-dependent in its use of vaccines in managing H1N1. |
| **Beck 2005** | To evaluate UK government officials’ failure to manage the BSE crisis effectively in relation to the nature of scientific information available, the handling and dissemination of that information, and the institutional framework through which key decisions were made. | UK | BSE | Grey literature; media reports | A centralised system in which government agencies control ‘science for government’ (i.e. through the use of government-friendly experts) with little public oversight is vulnerable to ‘capture’ by expert-interest group alliances. As a result, the potential for credible assessment and management of public health and safety risks is undermined. |
| **Behague 2009** | To explain why evidence-based policy making (EBPM) has thus far had a limited impact on the development and implementation of policy at the national and sub-national levels in developing countries. | Bangladesh, Burkina Faso, Ghana, Malawi and Nepal | Maternal and neonatal health | 52 semi-structured interviews with opinion leaders, policy-makers, clinicians, public health experts, and health system administrators in each country | The political contexts in which EBPM is played out promote uniformity of methodological and policy approaches, despite the fact that the public health literature repeatedly calls for disciplinary diversity. |
| **Bekker 2010** | To describe and explain from a sociological perspective how institutional arrangements for two-way research–policy interactions enable the co-production of ‘useable knowledge’ for ‘doable problems’ in health policy making. | Netherlands | n/a | Unclear | Many institutional arrangements function as boundary objects that allow for mediation between research and policy. This mediation occurs via both front stage and backstage processes. The backstage processes are an essential precondition for the co-production of evidence, policy advice and policy in the front stage. However, as a result of the increasing emphasis on evidence-based policy, and an accompanying instrumentalisation of research use in the policy process, some of the characteristics, and until now productive, elements of the Dutch system are threatened. |
| **Berridge 1996** | To provide a critical appraisal of the use of evidence in three historical cases of health policy in the UK. | UK | Drug use, alcohol policy, tobacco policy. | Policy documents | Powerful medical and civil servant ‘policy communities’ had a key role in linking evidence with policy on drug use.  The policy debate on alcohol was shaped by changes in the wider context of government policy-making, from a focus on social problems to an emphasis on the role of personal behaviour. |
| **Blume 2010** | To examine the way evidence is used in policymaking, relevant institutional developments, and the different meaning attached to different forms of evidence as a result of changes in the context for decision-making. | Netherlands | MMR vaccine | Archival material; scientific literature | Globalization of vaccine manufacture and the wider political background for health policy, including the process of European integration, have imposed a new logic of ‘what counts as evidence’ in immunisation policy making. In particular, there have been increasing pressures towards international standardisation and a growing reliance on international rather than national data. |
| **Bowen 2009** | To examine the relationship between evidence and policies on early childhood interventions. | Australia | Early childhood interventions | 35 interviews; policy documents; media reports | Important contextual factors in the use of evidence included government priorities, election promises and commitments, and risk assessment data about the implications of policy choices for winning, or retaining, votes.  The process of policy making was influenced by media pressure to act on child protection, understood as both a social good and an economic benefit, at a time of local elections. |
| **Broadbent 2012** | To reconstruct the debate around the HIV/AIDS Prevention and Control Bill in Uganda, with a focus on the ways in which evidence was used in that debate. | Uganda | HIV/AIDS | 28 semi-structured interviews; media reports; legal documents | Despite having access to documentation, the government failed to communicate the evidence base in support of the policy process. As a result, civil society had little opportunity to scrutinise or analyse anything other than the Bill itself.  Evidence from the numerous bodies responsible for data collection and analysis was used in instrumental ways to legitimate particular choices or claims. Some observers argued that the government manipulated statistics on HIV rates in order to ‘sell’ a success story of prevention and control.  Differing policy objectives (e.g. effective HIV prevention and control vs. protection of human rights) affected how and what evidence was used. |
| **Burchett 2012** | To explore processes of national decision-making around new vaccine adoption in seven developing countries and to understand the factors affecting these decisions. | Bangladesh, Cameroon, Ethiopia, Guatemala, Kenya, Mali, South Africa | Vaccine introduction | 95 semi-structured interviews with stakeholders | Some informants noted that local data on disease burden were considered important in driving the policy process. |
| **Court 2005** | To examine the use of evidence in the making of HIV/AIDS policy in three African countries | Uganda, Kenya, and Botswana | HIV/AIDS | Relevant literature; policy reports; 9 interviews; one discussion meeting | Governments that have demonstrated the most substantial policy responses to HIV/AIDS gained their legitimacy from delivering development results in other areas rather than from democratic processes.  The study raises concerns regarding the fact that a large share of the research on HIV issues is undertaken in or by external actors from high income countries, including concerns about the relevance of such research to developing country needs, the ease of access to findings, and the perceived legitimacy by local policy makers.  Other concerns include a lack of impact beyond the organisations directly involved in projects, hostility from recipient governments or suspicion about donor motives, and lack of support from traditional systems of authority at local level.  Political leadership (or the lack thereof) is an important variable in the policy process. |
| **Daniels 2008** | To explore how research information, in particular findings from randomised controlled trials and systematic reviews, informed policy making and clinical guideline development for the use of magnesium sulphate in the treatment of eclampsia and pre-eclampsia in South Africa. | South Africa | Use of magnesium sulphate in the treatment of eclampsia and pre-eclampsia | Interviews with local researchers and government officials. | The change to a democratic government in 1994 created a new governance model that was more open to the uptake of research findings. Academic researchers became more involved in policy making and were appointed to managing positions in the National Department of Health. |
| **De Vries 2006** | To provide a critical analysis of potential biases in evidence-based medicine. | Netherlands | Obstetric science | Unclear | Cultural and community values in the Netherlands have contributed to opportunistic selection of scientific evidence supporting the benefits of home-birth. |
| **Edström 2008** | To analyse and address the question of how policy is made for ‘children and AIDS’ in Cambodia | Cambodia | HIV/AIDS | Relevant literature; semi-structured interviews with stakeholders; one consultation meeting. | Political ammunition for policies to gain traction needs a locally relevant evidence base that engages both local leaders and development partners. However, local research seems to follow rather than lead policy changes initiated by international discourse and resources.  The cross-sectoral nature of the issue of children has provided particular challenges for establishing clear discourses and strong evidence in support of policies. |
| **Ensor 2009** | To understand why a government’s funded research report on maternal health influenced policy very quickly. | Nepal | Maternal health | Interviews with high-level officials, including ministers, and senior advisors. | Research had a powerful impact on policy making in combination with other factors, including the existence of good links between the principal investigator; and Nepal’s Minister of Health, effective dissemination strategies, and the fact that research findings were translated into a relatively straightforward message. In addition, the proposed policy was able to attract interests across the entire spectrum of the coalition government and was politically timely. |
| **Filtcroft 2011a** | To explore the use of evidence in the policy process leading to the implementation of a national bowel cancer screening programme. | Australia | Bowel cancer screening programme. | Document analysis; 34 semi-structured interviews. | Institutional barriers to the use of evidence were (1) the existence of institutional ‘silos’ and lack of cross-department communication; (2) confidentiality of advisory committee meetings, which prevents public scrutiny of the use of evidence; and (3) the timeframe and short duration of political mandates.  During an election period underlying tensions between stakeholders who control the selection of evidence for policy (experts, bureaucrats or external advisors) may be amplified.  In the heat of an election campaign, adherence to evidence may play a secondary role. |
| **Filtcroft 2011b** | To explore the unique resource, ethical, institutional and political environments in which the evidence was considered for the introduction of bowel cancer screening programme in New Zealand. | New Zealand | Bowel cancer screening programme. | Policy documents; 15 semi-structured interviews. | Issues of equity and social justice (i.e. civil rights of Maori ethnic groups) shaped the way in which individual and societal harms were valued. As a result, RCT evidence of the screening programme’s benefits – which satisfied policy makers in other countries such as the UK and Australia that adopted it – was considered insufficient in New Zealand.  While the prospect of imminent elections gave further incentive for cementing the health issue on the policy agenda, political pressure to act quickly further contributed to the abandonment of the evidence-based approach. |
| **Goede 2012** | To examine the use of epidemiological research for local health policy development in three municipalities in the Netherlands. | Netherlands | N/A | 129 semi-structured interviews with stakeholders; internet questionnaires; observations at meetings and review of documents | When scientific knowledge is in line with decision makers’ beliefs, values, responsibilities, and institutional interests, research is more likely to be incorporated in the policy process. |
| **Haas 2009** | To compare the experiences of seven industrialized countries in considering approval and introduction of the world’s first cervical cancer-preventing vaccine. | Australia, Canada, Denmark, Germany, New Zealand, Switzerland, and the U.S. | HPV vaccine | Reports of national cases. | The voice of special interest groups, including pharmaceutical companies, has been prominent in shaping the policy process in all countries.  The speedy introduction of a subsidized vaccination programme across a number of developed countries resulted from a convergence of interests, whether motivated by profit or public health needs. |
| **Hamel 2011** | To gain insight into factors affecting the knowledge translation performance of health professional associations in LMICs by describing the organizational elements and processes constituting capacity to use research, and examining the potential determinants of this capacity. | Burkina Faso | n/a | Review of documents; 6 semi-structured interviews. | The capacity of a (civil society) organisation to use and disseminate research is influenced by five organizational dimensions and processes, including organisational motivation and values, endogenous or exogenous stimuli (such as a change on government/health policy programme), organisational capacity to acquire, assess, and transform research (including the capacity to buy-in stakeholders), moderating organizational factors (such as the extent of dedicated human and financial resources). |
| **Haynes 2011** | To describe how policymakers use research or researchers in processes of decision-making and to examine how these activities relate to models of research utilisation. | Australia | n/a | 32 semi-structured interviews with civil servants, parliamentary ministers, and ministerial advisers identified as ‘research-engaged’ by public health researchers. | Issue polarization dictates the extent to which research or researchers are used technically or politically.  Researchers were engaged as persuaders and defenders when policy was strongly opposed and political imperatives were heightened; they were used as galvanizers, advisers, and clarifiers in circumstances where there was greater accord or, at least, when shared uncertainty and technical expertise had higher currency. |
| **Hughes 2007** | To assess the notion of ‘policy-based evidence’ to the development and implementation of the Illicit Drug Diversion Initiative (IDDI), a reform adopted in Australia in 1999 through the federal government’s ‘Tough on Drugs’ strategy. | Australia | Illicit drug use | Key stakeholders in the health sector, criminal justice sector, bureaucracy, non- government sector, and academia. | Scientific evidence was used selectively to support pre-existing national policy objectives |
| **Hunsmann 2012** | To examine political obstacles to evidence uptake by scrutinising the role of biomedical, epidemiological, and economic knowledge in the formulation of HIV prevention strategies in Tanzania | Tanzania | HIV/AIDS | 92 semi-structured interviews with domestic officials, bilateral and multilateral donor representatives, academic researchers or independent consultants and employees of Tanzanian, and international NGOs. | A number of contextual and political factors contributed to prioritisation of behaviour-centred HIV/AIDS prevention over structural approaches, including: (1) specialisation of implementation agencies; (2) donors’ preference for vertical programmes; (3) complexity and resulting issues of feasibility; (4) the expected timeline of political returns and policymakers’ preference for ‘rapid output’ over ‘deep impact’.  Cost-effectiveness analysis had a limited role in priority-setting processes. Most policymakers saw costing as an advocacy tool to legitimize the call for additional funding, not as a means to prioritise interventions and optimise resource allocation. |
| **Hutchinson 2011** | To understand the differential processes by which evidence on cotrimoxazole preventive therapy (CPT) has been taken up into national policy in three African countries. | Malawi, Uganda and Zambia | Cotrimoxazole prophylaxis -HIV/AIDS | 47 semi-structured interviews with stakeholders from government agencies, national and international nongovernmental organisations, multilateral agencies, research institutions, and hospitals. | In all countries, the structural and economic feasibility of implementing CPT (i.e. having a healthcare infrastructure and sufficient funds) was a central element for the adoption of research into policy, as well as the wider context of national HIV/AIDS plans (e.g. based on either prevention or bio-medical approaches). |
| **Iglesias 2005** | To compare the use of economic evaluations in decision-making process within the health-care systems in nine Latin American countries and three European countries. | Argentina, Brazil, Colombia, Cuba, Mexico, Nicaragua, Peru, Portugal, Spain, United Kingdom, Uruguay, Venezuela. | n/a | Surveys, systematic literature review (93 articles retrieved). | The establishment of ad hoc institutions for evidence-based health policy in some countries (i.e. UK and Portugal) was associated with increased use of economic evaluations. |
| **Jewell 2008** | To examine factors that may facilitate or hinder evidence-informed policy making in US state government. | US | n/a | 28 interviews with public service administrators and legislators. | States with fiscally conservative legislatures are less likely to enact some health-promoting policies, such as increases in the tobacco tax or coverage of dental care for children, despite evidence in support of these policies. On the other hand, some informants noted that the libertarian tradition of western states tends to create a baseline scepticism for any new regulations, as well as hindering the development of collective, multistate arrangements such as drug purchasing.  The pressure to make decisions quickly on a wide range of policies limits the legislators’ ability to accumulate much information about any one issue.  Lack of a research culture and research skills in policy-making is a barrier to evidence-based policy making. |
| **Jill 2006** | To examine how value judgments become embedded in decision-making processes towards the improvement of medical outcomes. | U.S. | Daily hemodialysis | Telephone interviews and e-mail correspondence with various stakeholders; policy documents. | Policymakers’ decisions to use currently available evidence to enact legislation or alternatively, to seek further evidence through additional research, reflects their valuation of social problems, respectively, viewed as being either worthy of immediate attention, or not. |
| **Klein 1990** | To examine issues and barriers in the use of evidence in the context of British NHS. | UK | n/a | Critical review of grey literature; academic studies. | Highly centralised policy making is likely to be less open to the uptake of research findings in comparison with de-centralised systems, as concentration of power tends to prevent pluralistic debate and thus make research input largely redundant. |
| **Lavis 2002** | To examine the use of research at three stages in the policy making process: prioritisation (or agenda-setting), policy development, and policy implementation. | Canada | n/a | Interviews; policy documents. | Issues other than evidence have been influential at various stages in the policy processes, including government’s interests and ‘policy legacies’ (meaning, for example, a policy that left one or more exceptional circumstance to be addressed at a later date).  ‘Content driven’ decisions may be more amenable to the influence of research in instrumental (e.g. specific and direct) ways than are large-decisions concerned with, for example, jurisdictional considerations. |
| **Liu 2006** | To document the authors’ experiences in addressing critical questions for converting research results into policy actions concerning health insurance. | China | Health insurance for rural populations. | Personal narrative | In countries where free, independent research is lacking, research input for policy change is more likely to come from studies conducted by international institutions.  Research findings on the link between health and rural poverty attracted the interest of policy-makers as they related to the wider policy drive towards economic growth.  The research to policy process was also successful as a non-confrontational approach was sought to persuade policy makers, giving leaders time to prepare a timely response.  Other key factors influencing the uptake of research were the use of national data and indicators, and the development of policy options that were tailored to the local context. |
| **Milewa 2005** | To provide a critical appraisal of evidence-based health policy by drawing on a study of decision-making processes at the National Institute for Health and Clinical Excellence (NICE). | UK | n/a | 33 interviews; observations at professional meetings. | Even the most ‘objective’ procedural and methodological approaches to the appraisal of health technology may be influenced by the aims, strategies and power of participating individuals and actor-groups. |
| **Moodley 2000** | To present results of a case study of research utilisation and explore the potential for reducing the gap between research and policy action. | South Africa | Nutrition policy | Literature review; semi-structured interviews with key informants. | With the transition to a democratic government after the end of apartheid, new institutions for health system research were created, supported by the new government and international donors.  Newly elected governments are under pressure to perform. Research that demonstrates visible public benefit is thus more likely to be taken up. |
| **Mubyazi 2005** | To analyse the way in which evidence on antimalarial drug policy change was produced and used in Tanzania. | Tanzania | Malaria | 39 interviews with health professionals, researchers, and senior officers; documents review. | Institutional/political barriers to evidence-based policy making included commercial interests of drug manufacturers and fear of financial implications of policy change.  Implementation of the same policy in neighbouring countries contributed to acceptance from decision makers. |
| **Nattrass 2008** | To review the political context of AIDS policy in post-apartheid South Africa. | South Africa | HIV/AIDS | Interviews with high-level policy makers and ministers | Various political and institutional factors may have influenced the use of evidence and the rejection of scientific consensus, including the political struggle between the government and civil society, the quest for African national identity as opposed to ‘Western’ science and commercial interests, and the lack of autonomy of domestic institutional bodies charged with scientific assessment. |
| **Northington 2006** | To examine the relationship between scientific research and policy on health inequalities in the U.S. and the factors increasing the impact of research on policy. | U.S. | Health inequalities; racial and ethnic discriminations. | Policy reports. | Differing mandates of institutions charged with scientific assessment are reflected in the writing and framing of scientific reports they produce.  Framing narratives in scientific reports had a key effect on policy processes. |
| **Orem 2012** | To elaborate a middle-range theory of knowledge transfer in Uganda that can also serve as a reference for other low- and middle income countries. | Uganda | n/a | Interviews; databases of journals | Institutional barriers to knowledge transfer included lack of frameworks and structures to link researchers and policy makers from the start of the research to policy process and the inability of policy makers to understand, process and use research effectively. |
| **Pappaioanou 2003** | To evaluate the use of a strategic framework to enhance capacity for evidence-based public health in developing countries, called Data for Decision Making (DDM). | Bolivia, Cameroon, Mexico, Philippines. | n/a | Interviews | The existence of a talented, visionary, and strongly motivated senior health official who championed DDM concepts was essential for country ownership of DDM goals, objectives, and activities. |
| **Parkhurst 2012** | To examine framing mechanisms underlying the support to or criticism of PEPFAR’s ‘ABC policy’ (Abstain, Be faithful, use Condoms) for HIV/AIDS control in Uganda. | Uganda | HIV/AIDS | Interviews with high-level civil servants and advisory bodies; academics and members of key think tanks and advocacy groups. | Core beliefs and moralities may contribute to selective interpretation of evidence that supports such beliefs.  Given the lack of definitive evidence, research findings may be open to interpretation by commentators with differing moral and/or political agendas. |
| **Pittman 2006** | To better understand the complex relationship between health equity research and health policy. | USA, UK, Netherlands, China, South Africa, and Chile. | Health equity | Unspecified | In high-income countries, greater attention was paid to the professional legitimacy of institutions, individuals and journals, while in developing countries the existence of trusted personal messengers appeared essential.  In China, the notion that out-of-pocket spending on health care is a major cause of poverty was an especially effective argument in driving the policy process. |
| **Rennert 2003** | To explain variations in policy on breast cancer screening across countries despite the large, common and widely accepted international literature on its effectiveness. | Israel, USA, UK, Netherlands, France, Germany | Breast cancer | Policy documents and scientific reports. | Public opinion and acceptance of interventions was an important factor in determining the choice of particular interventions. In the U.S., vested interests had a critical role. |
| **Saguy 2005** | To examine claims and claimants involved in the contemporary obesity debate in the U.S. and the way different groups framed the issue. | U.S. | Obesity | Documents; participant observations; 21 semi-structured interviews with stakeholders. | Different interest groups may use evidence selectively to establish their own credibility or discredit their opponents.  Notions of morality may play a central role in many medical disputes, and scientific arguments can be used to stymie rights claims and justify morality-based fears. |
| **Salvatella 2000** |  | Uruguay | Chagas disease; foot and mouth disease. | Literature review; personal narrative. | With the restoration of democracy in Uruguay after 1984, a new environment for evidence-based health policy was created, as new funding was allocated to science, many researchers returned from exile, and segments of the population whose health needs and concerns had formerly been ignored, finally received attention. |
| **Schneider 2002** | To provide an overview of the policy debate on HIV/AIDS in South Africa, with a focus on the politics of President Mbeki’s controversial policy and the role of other stakeholders such as civil society organisations. | South Africa | HIV/AIDS | Review of various documents, media releases. | President Mbeki’s rejection of ‘Western’ biomedical consensus on HIV/AIDS can be associated with political issues of national identity and power struggles between central government and civil society organisations. |
| **Schwartz 2004** | To provide an analysis of political factors influencing evidence-based health policy-making. | Israel | Health reform (1995) | 18 interviews with stakeholders. | Use of data varies between issues depending on the level of politicisation of the issue and whether there are implications for increases in the budget.  Authors distinguish between first order decisions (whether to do something) and second order decisions (how to do it; the details). Data are more likely to be used for second order decisions.  First order decision which are technical in nature and do not involve ‘turf wars’ between departments or policy actors are also likely to be informed by data than more politicised first order decisions.  Differences in the use of data exist also between the financial and service level (high data use) and that on the effectiveness and quality of care (almost no data use). |
| **Smith 2012** | To explore how the organisation of policy-making bodies shapes the relationship between research and policy. | UK | Health inequalities | 62 semi-structured interviews and review of 59 policy statements. | Policy-making institutions in the UK have operated as filters for ideas about health inequalities, encouraging the influence of ideas that are in keeping with overarching ‘policy paradigms’.  Responsibilities are divided within bureaucratic organisations in such a way that individual civil servants are compelled to focus on small, specific areas of policy activity, making it extremely difficult for them to engage with ideas beyond their immediate area of responsibility.  A lack of institutional memory within policy institutions enables similar ideas to be regularly recycled, creating the illusion that research is informing policy far more than it is. |
| **Smith 2007** | To examine the use of evidence in policy debates over health inequalities in the UK. | Scotland, England | Health inequalities | 58 interviews with academics and non-academic health policy makers. | Limited support found for the political model of evidence use (research used to support predetermined political objectives) and the tactical model (research used to delay decision making).  The ‘sellability’ of ideas was shaped by the wider political framework. If an idea is thought to overtly conflict with ruling political ideology, marketing to a policy audience may require a shift in meaning of the idea or, at the very least, a more flexible construction of the idea. |
| **Sumner 2008** | To provide a comparative analysis of the ‘market for evidence’ in child health policy | India, Vietnam | Child health policy | 55 interviews with senior policy makers and researchers. | The nature of the political system (e.g. democratic or autocratic) is not necessarily a key factor in influencing the use of evidence in policy making.  Orientation of external donors influenced evidence use.  Level of consensus on an issue determines evidence use. Evidence use is less likely where everybody ‘knows’ (agrees on) what the issue at hand is. |
| **Thomson 2007** | To examine the ways in which evidence was used by parliamentary politicians during the debate over the effects of second-hand smoke and smoke-free policy in New Zealand. | New Zealand | Second-hand smoke | Parliamentary records, media, and relevant databases. | The tobacco industry and its allies have been successful in persuading a proportion of national level politicians that there was a significant doubt about health risks of second-hand smoke.  Evidence alone is not sufficient, but powerful narratives are needed to drive the process of policy making  Ideas may influence the use of evidence; for example some politicians attacked smoke free policy because it restricted personal freedoms. |
| **Tomson 2005** | To discuss various factors influencing decision-makers’ perceptions of health system research for the implementation of the Lao National Drug Policy. | Laos | Essential Medicines. | 90 structured and semi-structured interviews. | The process of evidence-based policy was relatively straightforward because potential opponents (such as pharmacists and doctors) were not organised to lobby effectively against the policy. Also, multinational drug industries were probably not interested in the small Lao market.  Some respondents were not able to distinguish between research results and information in general. In Lao, ‘research’ is a relatively new concept that is often used in relation to the review of documents or events. |
| **Trostle 1999** | To report the results of a descriptive study of the relationship between health research and policy in four vertical programmes in Mexico (AIDS, cholera, family planning, immunization). | Mexico | Family planning, immunisation, AIDS, cholera | 67 interviews with researchers and officials from different levels of government and administration | Evidence-based policy is impeded by a political culture in which decision making is based on experience and immediate pressure rather than research and strategic planning prioritisation.  Further barriers include lack of institutional mechanisms to manage evidence use, insufficient technical expertise amongst bureaucrats, the influence of powerful lobbies, especially financial groups, and reluctance to endorse controversial research (e.g. research associated with sexuality).  Centralisation contributes to hierarchical management of information. This sometimes means that research results do not arrive at operational levels, where they could have greater impact and usefulness. |
| **Tulloch 2011** | To examine comparatively case studies of research utilisation in policy and practice for sexual and reproductive health. | Ghana; South Africa; Tanzania | Sexual and reproductive health | Observational study | Institutional and political structures are vital in influencing the willingness of policy actors to respond to new research findings. Insufficient guidance on implementation, cultural issues (e.g. religious beliefs associated with male circumcision) and lack of commitment are also important factors, as well as personal links between researchers and policy makers. |
| **Uneke 2011** | To present and discuss policy makers’ perceptions of capacity for the use of evidence at individual and organisational levels. | Nigeria | n/a | Cross-sectional survey of stakeholders’ perceptions. | The presence of competent researchers in organisations is crucial as they can act as facilitators of knowledge use and train colleagues in appropriate skill.  Organisational incapacity can thwart the impact of even highly skilled and highly motivated individuals.  It is vital to change organisational culture and create clear strategies that encourage evidence use, as well as communication channels for exchange of ideas and expertise. |
| **Van Kammen 2006** | To describe a recent successful experience with knowledge brokering in the Netherlands and discuss the requirements for effective institutionalisation of this approach and its potential to assist health policy development in low-income countries based on the experience of the Regional East-African Health (REACH)-Policy Initiative. | Netherlands/ Kenya, Uganda, Tanzania (REACH Project) | Fertility (IVF) policy (Netherlands); REACH Project | Unspecified | Knowledge-brokering should be embedded in institutional structures for evidence-based decision-making. |
| **Waddell 2005** | To examine the use of evidence in policy development on antisocial behaviour in children. | Canada | Children’s mental health policy and conduct disorder | 32 semi-structured interviews with politicians and senior civil servants. | Research evidence was valued and used in policymaking, but just as one source of ideas and information among many. Barriers to the use of evidence included institutional constraints (and particularly the fragmentation across federal, provincial and local levels of government) and competing interests of different stakeholder groups concerned about child antisocial behaviour.  The use of research evidence in policymaking could be enhanced if researchers learned about the competing influences on the policy process, formed research-policy partnerships, and engaged in public debates.  Emotional public reactions to child antisocial behaviour compelled front line service providers to make swift and tough decisions. By contrast, federal policy makers could respond ‘more contemplatively’ because of their distance from the front line. |
| **Wilson 2006** | To describe a rare example of explicit policy commitment to follow the findings of a systematic review on the efficacy and safety of the fluoridation of drinking water. | UK | Water fluoridation | Document analysis | Even when a systematic review is sustained by regular interactions between researchers and policy makers and is conducted ‘to order’ - explicitly to inform policy - it may not be influential if its findings contradict prior beliefs and policy intent of key decision makers. |
| **Wimbush 2005** | To discuss examples of successful collaboration between research, policy and practice communities in two pilot projects. | Scotland | HIV/AIDS prevention for IV drug users/ School-based sexual health education (SHARE) pilot projects | Unspecified | Specific aspects of post devolution settlement in Scotland have affected evidence-based health policy and practice (i.e. interactions between research, policy, and practice communities; greater political scrutiny; political will to challenge wicked problems).  Geographical dispersal of government agencies has made networking and collaboration logistically harder.  Politically sensitive nature of HIV/SHARE pilot schemes was such that research evidence, perceived as ‘neutral’ or independent was crucial in justifying their launch. |
| **Wolf 2007** | To provide a critical review of the scientific and cultural bases of a campaign on breast-feeding in the U.S. | U.S. | Breastfeeding | Scientific articles, press releases, newspaper articles. | The campaign on breast-feeding capitalised on public misunderstanding of risk and risk assessment by portraying infant nutrition as a matter of safety versus danger, thus creating spurious analogies. The campaign also exploited deep-seated normative assumptions about the responsibility that mothers have to protect babies and children from harm.  Scientific evidence can be manipulated more easily when randomised control trials are not a viable option, as is the case of infant-feeding research. |
