# Supplementary material for: Political and Institutional Influences on the Use of Evidence in Public Health Policy. A Systematic Review
Source: PLoS One. 2013 Oct 30;8(10):e77404. doi: 10.1371/journal.pone.0077404 (PMC3813708; doi:10.1371/journal.pone.0077404)
Supplement: Text S1 — Research protocol. (DOCX) [file pone.0077404.s003.docx]

**Protocol for systematic review.**

**Political and institutional influences on the use of evidence for public health policy.**

The GRIP-Health project investigates the use of evidence in public health policy, with a focus on political and institutional factors influencing the process of research utilisation. In the first phase, we will conduct a systematic review of the literature on this issue with a view to developing a conceptual framework that will inform the analysis of six country case studies in the second phase. The systematic review aims to synthesise what is known about:

1. the influence of key features of different political systems on the use of evidence for health policy;
2. the influence of institutional mechanisms and processes on the use of evidence for health policy;
3. the influence of other contextual factors that may contribute to the the politicisation and contestation of health evidence.

**Search Strategy**

Electronic search strategy

Relevant material will be identified first through electronic search strategies. The following electronic databases will be searched from start date (but no earlier than 1990) to 2012:

- Global Health;
- Healthcare Management Information Consortium (HMIC);
- International Bibliography of the Social Sciences (IBSS);
- MEDLINE;
- PubMed;
- Social Policy and Practice;
- ISI Web of Science
- The British Library

In addition, a number of websites will be searched for grey literature, including the online repositories of the Program in Policy Decision Making at McMaster University, the Centre for Evidence & Policy at King’s College, the WHO Evidence into Policy Network (EVIPNet), and Evidence to Policy Initiative (E2Pi) at the University of California at San Francisco. Internet search engines such as Google and Google Scholar will also be used. The latter are useful tool for searching material not published in journal or available online such as monographs and book chapters.

The following basic search strings will be used for electronic databases:

1. Health polic* AND (research OR evidence) AND (governance OR institution* OR polit*);
2. Evidence-based AND (health* OR medicine) AND policy AND (governance OR institution* OR polit*)

A ‘search diary’ will be maintained detailing search results and progress. Titles and abstracts of studies to be considered for retrieval will be recorded on an Endnote library, along with details of where the reference was found. Retrieved studies will be filed according to inclusion/exclusion decisions. Checks for duplicates will be performed after data collection. References will then be transferred to a Mendeley database to facilitate collaborative working during the screening and data extraction phases.

Manual search strategy

We will also conduct follow-up searches on citations found in other studies and we will be seeking information from experts. Journals that seem particularly relevant will be hand-searched. In both electronic and manual searches, the initial selection criteria will be broad to ensure that as many studies as possible are identified and their relevance to the review assessed. In the first instance, we will screen the title and the abstract of the articles generated and any that are obviously unsuitable will be excluded. Articles which have passed this screening phase will be read in their entirety and the decision to exclude or include them will be made on the basis of the selection criteria set out below.

Inclusion criteria

- must include empirical material;
- must document data sources;
- must focus on the use of evidence/research in policy process;
- must focus on health;
- must include some analysis of the influence of key features of political systems and/or institutional mechanisms and processes on the use of evidence
- AND/OR must include some analysis of the influence of other contextual factors on the politicisation and contestation of health evidence.

Exclusion criteria:

- articles published earlier than 1990, as the ‘evidence based medicine’ movement, from which current calls for evidence informed policy in health emerged, is widely seen to have taken shape around this year;
- articles that examine knowledge uptake in the private sector.

**Data extraction, tabulation and categorisation**

Data will be extracted and tabulated by using a uniform data extraction tool. In addition to information on the study characteristics (e.g. objectives, methodological approach, country focus, health issues), relevant findings will be identified and analysed according to three research questions derived from political studies theories and designed to address the gaps identified above in the existing literature on evidence to policy processes
